# Mental Health Applications of Generative AI and Large Language Modeling in the United States

**DOI:** 10.3390/ijerph21070910

**Published:** 2024-07-12

**Authors:** Sri Banerjee, Pat Dunn, Scott Conard, Asif Ali

**Affiliations:** 1 School of Health Sciences and Public Policy, Walden University, Minneapolis, MN 55401, USA; 2 Center for Health Technology & Innovation American Heart Association, Dallas, TX 75231, USA; pat.dunn@heart.org; 3 Converging Health, Irving, TX 75039, USA; scott.conard@converginghealth.com; 4 McGovern Medical School, University of Texas Health Science Center at Houston, Houston, TX 77030, USA; asifalitex@gmail.com

**Keywords:** mental health, ChatGPT, large language modeling, predictive analytics, prevention, diagnostic accuracy

## Abstract

(1) Background: Artificial intelligence (AI) has flourished in recent years. More specifically, generative AI has had broad applications in many disciplines. While mental illness is on the rise, AI has proven valuable in aiding the diagnosis and treatment of mental disorders. However, there is little to no research about precisely how much interest there is in AI technology. (2) Methods: We performed a Google Trends search for “AI and mental health” and compared relative search volume (RSV) indices of “AI”, “AI and Depression”, and “AI and anxiety”. This time series study employed Box–Jenkins time series modeling to forecast long-term interest through the end of 2024. (3) Results: Within the United States, AI interest steadily increased throughout 2023, with some anomalies due to media reporting. Through predictive models, we found that this trend is predicted to increase 114% through the end of the year 2024, with public interest in AI applications being on the rise. (4) Conclusions: According to our study, we found that the awareness of AI has drastically increased throughout 2023, especially in mental health. This demonstrates increasing public awareness of mental health and AI, making advocacy and education about AI technology of paramount importance.

## 1. Introduction

The age of technology colliding with the advances in medicine has resulted in a renaissance of diagnostic and treatment modalities for various disease conditions [1,2]. More specifically, artificial intelligence (AI), especially large language modeling (LLM), is in its heyday, with influences in many medical disciplines including population health, cardiovascular health, neurological health, and mental health [3,4,5,6,7]. The shortage of mental health practitioners has required novel technologies like AI. Due to the shortage of mental health workers, there is increased burden on primary care physicians to treat mental illnesses, although they are not equipped to handle these, which traditionally require a referral to a specialist. This necessitates the innovative use of technology to address shortages in the healthcare profession.

There are novel generative AI tools, such as Google Bard (LaMDA), ChatSonic API, Microsoft’s GPT-3, and Facebook’s Robustly Optimized BERT Pre-training Approach (RoBERTa), that have the potential to alleviate some of the mental health provider shortages. One of the original generative AI tools, ChatGPT (Chat Generative Pretrained Transformer), represents a revolution in the world of artificial intelligence. OpenAI, debuted in 2020, advanced this conversational AI technology powered by a linguistic model known as GPT, short for Generative Pre-Training Transformer [3]. The ChatGPT-3 model was the latest in a line of large pretrained models designed for understanding and producing natural language [1,2]. In recent times, society has been gripped with awe about the potential that this new technology brings to the area of mental health.

One area of application that researchers have touted as successful is the improvement of diagnostic accuracy of various disease conditions. The discipline of mental health is no exception. For instance, one study showed how large language modeling or generative AI can be harnessed to make mental health prediction and prevention through retrieving online text data [8]. Additionally, more recently, along with improved treatment, there is a drive to screen and prevent mental illnesses in healthy individuals living in vulnerable populations.

Many people learn about medical conditions by relying on using Google and other search engines. There are well-researched and efficacious applications in the fields of cardiology, radiology, and surgery, among other areas of medicine. Due to the technical nature of LLM, there is trepidation in applying the novel technology to medicine. Over recent years, as social media has become a source of medical information, researchers found that analyzing user-generated data can improve the effectiveness of AI and increase access to medical information by the public [8]. Even Twitter data have been used for LLM-driven sentiment analysis and can contain vital information to diagnose depression and even suicidal ideation. Many social media users often express their perspectives and thoughts of depression and suicide on these platforms.

There are other applications of user-generated data in mental health through the applications of AI. LLMs can be useful in predicting mental health disorders for leveraging natural language processing for parsing user-generated information. Some researchers utilized online social media datasets with high-quality human-generated mental health labels. Reddit was their platform of choice because it has nearly half a billion active users who discuss a wide range of topics [9]. The posts and comments are publicly available, and the researchers could collect data going back to 2011. This vast availability of user-generated data can be used to create more precise AI models.

In many countries and certain areas within the United States, there is fewer than one psychiatrist per every 100,000 population. Tutun et al. found [10] that AI can be used to create decision support systems to accurately predict mental health disorders as indicated in the Diagnostic and Statistical Manual of Mental Disorders (DSM-5) and International Classification of Diseases. Furthermore, there is still room for artificial intelligence applications in mental health. For instance, typically, primary care physicians are not trained in mental health, necessitating further collaboration. AI offers a lot of promise in applying, appreciating, and embracing the application of mental health. More specifically, AI has revolutionized how magnetic resonance imaging (MRI) allows for improved diagnosis through providing higher-resolution images and potential biological changes that are connected to depression [11]. Other instances of AI application include diagnosing sepsis in premature babies from metrics such as vital signs and monitoring devices [12,13]. Even though in medicine there are many examples of AI applications, there is not a thorough understanding of how artificial intelligence has impacted the field of mental health.

Globally, mental health problems affect one in eight individuals [12]. Mental health is strongly connected to physical health, with large swaths of people becoming aware of the importance. Khubchandani et al. found [14] that depression is strongly intertwined with diabetes as it relates to poor outcomes. Banerjee et al. found [15] that social isolation, a byproduct of mental health, is also a predictor of overall mortality. One distinct way to diagnose mental health disorders is the 90-question Symptom Checklist-90 (SCL-90-R). Tutun et al. [10] found a way to leverage AI to address problems in diagnostic methods. However, the number of questions and the complexity of the SCL-90 questionnaire necessitates alternative AI-driven ways to maintain mental health diagnostic accuracy, even after the reduction in the number of questions, for instance, decreasing a 90-item questionnaire to a 28-item questionnaire (SCL-20-AI) without any human input, to an accuracy level of 89% [10]. Additionally, the researchers emphasized the importance of establishing close cooperation between the creators of AI-based decision support systems and mental health practitioners.

Many treatments of depression and anxiety have proven to be ineffective against mental health disorders, due to which lack of medical adherence is a concern. Treatment for mental health disorders is only about 30% effective [16]. The lack of efficacy is due to the lack of understanding, requiring the use of more sophisticated algorithms. There are many other reasons for the lack of effectiveness of treatment, including stigma and lack of access. Artificial intelligence can integrate genetic heterogeneity and other considerations to utilize the concept of personalized medicine, to create tailored treatment towards the individual.

Depression and anxiety remain major mental health and public health problems that are on the rise. In 2023, Canady et al. found [17] that there were approximately 29% of the population that have mental health problems. This increased by 10 percent from the year 2015. Staggering statistics have paved the way for novel technologies such as AI, a more comprehensive treatment modality. Additionally, according to a Gallup Panel [18], the 17.8% who either had a diagnosis or currently have been treated for depression is up from 2015. At the same time, the cost of depression has skyrocketed. In order to stem the tide of increasing mental health disorders, improved diagnostic methods, through generative AI, have proven to be effective.

According to the Substance Use and Mental Health Service Administration (SAMHSA), mental illness and substance use disorder treatment spending from all public and private sources was USD 280.5 billion in 2020, an increase from USD 171.7 billion in 2009 [19]. Major depressive disorder is now considered the leading cause of disability worldwide. However, in the discipline of mental health, there is a high rate of medication nonadherence. Medication nonadherence is higher among people with mental health disorders than chronic physical conditions. Additionally, chronic diseases may need to be addressed after successfully treating mental health conditions.

One of the hurdles that mental health professionals have to grapple with is the lack of adherence to medications. There are many reasons for medication nonadherence, such as fear of potential side effects, lack of disease acceptance, and lack of awareness of not taking the medication regularly. Also, polypharmacy due to comorbid chronic diseases can be another deterrent for medication adherence due to lack of understanding or poor patient–physician communication. Additionally, the lack of current efficacious mental disorder treatments leads to higher nonadherence rates. However, the consequences of medication nonadherence can have far-reaching consequences. For instance, there may be an increase in relapse or even exacerbation of mental health symptoms or even suicidal tendencies.

With the lack of efficacy and adherence to mental health medications, AI technology has become an integral tool to better understand the consequences of mental health and create more targeted therapies [20,21]. While the application of artificial intelligence is not new, the rapid spread of generative AI and large language modeling is recent and has immense potential in diagnosing and treating mental health conditions [22]. In fact, the potential of mental health is seemingly limitless and broad in scope. What is not known is how much interest there is for generative artificial intelligence, particularly around mental health.

Mental illness continues to be a very serious public health problem. Ivanov et al. found that the traditional methods espouse a very simplistic biological approach to treat mental illness, with the use of psychotropic drugs that are not very effective [23]. However, new approaches are important to develop with the increase in the prevalence of mental health conditions and a shortage of mental health practitioners, importantly in resource-poor settings [24,25]. Large language models can be harnessed to create inexpensive tools that can be used to address these shortages [26,27]. With the aid of generative AI, complex treatment modalities can be used to better address psychiatric conditions. While most studies focus on the medical context of mental illness, what we investigated is how the popularity of AI in mental health is a reflection of AI literacy in the general public, especially in the area of mental health.

## 2. Methods

In this study, we used Google Trends, and, through search patterns on the Internet, we analyzed the web queries that were made in the search engines, such as the Google website search engine. All the searches were conducted within the United States. To conduct analyses, we downloaded the data for all searches in the United States.

After downloading the output, we conducted further analyses. We used the portal to determine the proportion of searches for the terms “AI”, “AI and Depression”, “AI and Anxiety”, and “AI and Mental Health” over the time series of from 1 January 2023 to 31 December 2023 among all searches performed on Google Search and found a relative search volume (RSV) index. AI was the proxy used for multiple terms (generative AI, artificial intelligence, ChatGPT and large language modeling). Google Trends provides a list of topics—these are a group of search terms that fit into the same general concept. The most important topics were “ChatGPT”, “Bard”, and “Generative artificial intelligence”. The RSV is the query share of a particular term for a given location and time, normalized by the highest query share of that. Sample data are used to display interest in a search term on a global, national, or city level. The search queries are normalized on a scale from 0 to 100 in order to compare search data. Search data are presented using an RSV, where 100 indicates the peak of search volume. Google Trends also normalizes the data based on the time and location of a search query.

We used the Box–Jenkins time series modeling estimation method to determine what the search volume of “AI and mental health” will be by the end of 2024. Autoregression (ARIMA) and other statistical analyses were run using SAS v.9.4. Diagnostics for the four models were completed checking for autocorrelation. The adequacy of the model was tested using chi-square test statistics for white-noise residuals. Our autoregressive integrated moving average model is robust to time series biases, such as recurring periodicities. Additionally, a trend line for the time series plot with additional Lowess estimators plot, a nonparametric regression, was generated. Here linear methods do not perform well. Lowess fits a regression line through the moving central tendency of “AI” along the time gradient. Additionally, using ArcGIS, we geospatially analyzed the County Health Rankings 2023 to assess the distribution of mental health workers per 100,000.

## 3. Results

**Figure 1:**
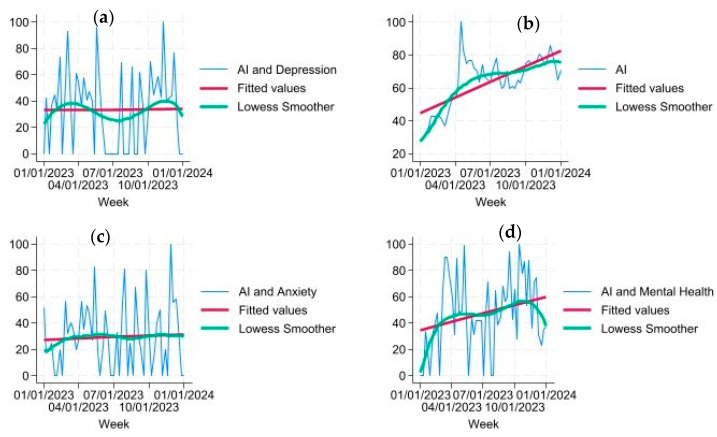
Each graph includes the actual RSV, the fitted values, and the Lowess smoother. (Top left (**a**)): This graph represents the relationship between RSV versus time for “AI and Depression”. (Top right (**b**)): This graph represents the relationship between RSV versus time for “AI”. (Bottom left (**c**)): This graph represents the relationship between RSV versus time for “AI and Anxiety”. (Bottom right (**d**)): This graph represents the relationship between RSV versus time for “AI and Mental Health”.

According to Table 1, each of the search terms reached a maximum at varied times in 2023. For instance, the term “AI” steadily increased from January onward, according to Figure 1b. This aligns with the findings in Figure 1, showing that the term “AI” reached its maximum by April. The term “AI and Mental Health” reached the maximum RSV in October, demonstrating a more gradual increase. The RSV for the terms “AI and Depression” and “AI and Anxiety” reached the maximum in November.

As seen in Figure 1, the RSV for the year 2023 varied according to the search volume in mental health and AI. According to Figure 1b, the term “AI” was already popular, and the trend line demonstrates that by the end of 2023, the search for this search term had drastically risen throughout the year. The next terms searched along with AI demonstrated further variations. The term “AI” was tested with “mental health” (Figure 1d), “anxiety” (Figure 1c), and “depression” (Figure 1a) individually to determine search term volume. In Figure 1d, “AI and mental health” demonstrates an increasing trend; however, the starting RSV was slightly lower than “AI” alone. Figure 1c,d demonstrate a steady trend with no change in RSV between the beginning of the year and the end of the year.

In Figure 1, the Lowess smoother showed variations present in the trend for each RSV through time that were not quite linear—as was seen in each of the search terms. Unlike Figure 1d “AI and mental health”, both “AI and depression” (Figure 1a) and “AI and anxiety” (Figure 1d) remained steady throughout the year 2023. Also, as seen in Figure 1a, the Lowess smoother seemed to have a seasonal variation throughout the year.

**Figure 2:**
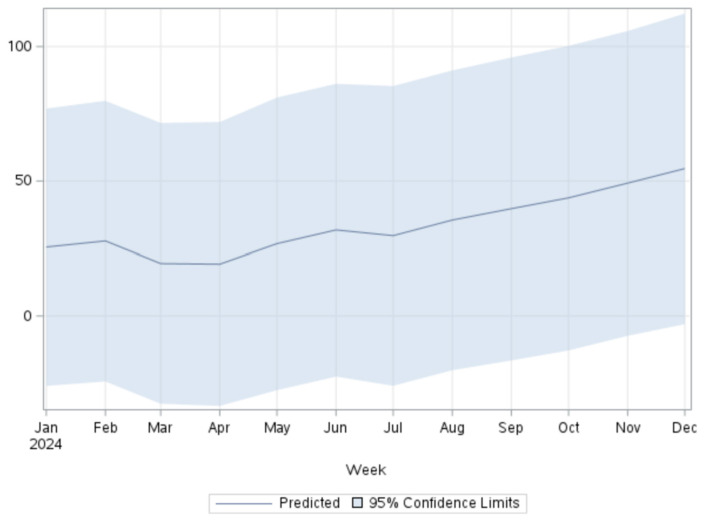
Predicted RSV for the terms “AI and mental health” in 2024 using the ARIMA model.

According to Table 2 and Figure 2, the projected RSV through December 2024 is expected to increase from 25.5 to 54.6, indicating a 114% increase throughout the year. This aligns with the findings from Figure 1d, where an increase is already indicated in the year 2023.

**Figure 3:**
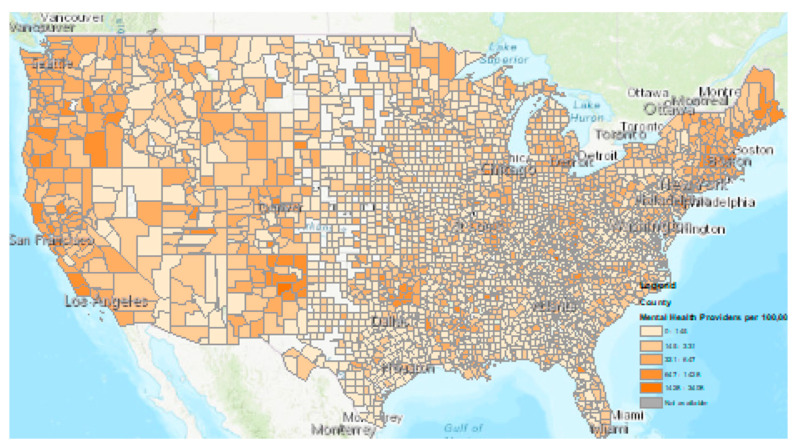
Distribution of mental health providers per 100,000. Light blue and white are areas with mental healthcare shortages.

Finally, according to Figure 3, we conducted a geospatial analysis and observed that while on a national level there is a shortage, there are variations and severe shortages in the distribution of mental health workers per 100,000. Some of the rural counties in the United States did not have a mental health professional at all, as indicated by white and lighter blue. Darker blue indicates an adequate number of mental health workers in 100,000.

## 4. Discussion

In recent times, society has been gripped with curiosity about the potential that this new technology of generative AI has for use in the discipline of mental health. In fact, the potential in mental health is seemingly limitless [27]. According to our study, a novel finding was that the search volume and awareness of artificial intelligence increased by 257% from January to April of 2023. As more mental health datapoints will become available, generative AI will be leveraged and harnessed to improve many mental health discoveries and diagnostic methods [28,29,30]. The discipline of mental health is no exception in helping unleash the potential of AI. The increase in Google searches related to AI and mental health between January 2023 and December 2023 may reflect increasing public recognition of the clinical aspects addressing them. More specific terms relating to depression and anxiety as they relate to AI are not as frequently searched but alowly increasing in popularity. Healthcare practitioners should use plain English terms for online discovery of AI-driven mental health resources and be aware of health literacy.

One way to increase awareness is to educate patients about the techniques and the applications of AI. Medical chart abstraction is a method that has gone through multiple iterations and has gone through a major LLM breakthrough in the discipline of mental health. Through linguistic diversity, ChatGPT-4 has previously exhibited enhanced multilingual abilities to apply linguistic machine learning algorithms to electronic health records to detect “suicidal thoughts” or “suicide attempt” [31,32]. Other words that have been used for mental health chart abstraction include “anti-depression medication” [33,34,35]. Moreover, research also shows that social media, with the help of AI, can predict diagnoses in medical records more accurately than self-report surveys [35,36,37]. However, a combination of multiple AI-driven diagnosis methods can be more accurate than what physicians can achieve alone.

Another novel finding in our study was that the interest in AI in mental health has been increasing throughout 2023 and is expected to increase by 114% throughout the year 2024. This shows that awareness will increase over time for public health and mental health practitioners alike. In comparison, we found, through geospatial analysis, that there is a shortage in mental health providers across the country, necessitating the importance of AI-guided mental health services. More and more people are relying on ChatGPT and generative AI to receive medical advice in lieu of a physician’s advice [38]. Additionally, AI can unfold the possibilities of applications in mental health treatment.

AI technology can provide decision-making support that healthcare practitioners can act on. This can aid primary care providers to rely on AI technology to further guide treatment in mental health, alleviating some of the burden experienced by general practitioners. AI can not only improve diagnostic accuracy in many ways, but the technology can also aid in treatment. For diagnosis, researchers found that AI mental health solutions such as wearables can interpret bodily signals using sensors to offer help, instead of waiting for a user to interact. For instance, an individual that is experiencing an anxiety attack can use physiological changes to become aware of these changes to tailor AI-guided treatment for each individual. Utilizing sleeping patterns, physical activity, heart rate, and rhythm variability AI can be used to assess the user’s mood and cognitive state. AI-generated data can be joined with therapy session transcripts to improve treatment quality. Creating specialized AI-generated algorithms can improve treatment quality and efficacy. Additionally, chatbots can provide timely and personalized interventions [39].

One caveat of our findings is that due to AI being in the news so frequently, some of the peaks in search volume were attributed to news announcements. For instance, as of the writing of this article, US and China agreed to create an infrastructure to map out a framework for developing AI responsibly, creating an increased level of interest in the topic area [40]. Using the Levels of Emotional Awareness Scale (LEAS), Elyoseph et al. found that ChatGPT-3.5 demonstrated an exceptional ability to differentiate and elucidate emotions from textual cues, outperforming human sample norms [24]. This can lead to the AI-driven administration of cognitive behavioral therapy (CBT) according to the emotions detected in the patient.

Mental health therapists have mixed opinions about the usage of AI in conducting therapy sessions. In 2024, the American Counseling Association (ACA) has an AI Working Group that they have convened in prioritizing client wellbeing. However, the ACA has warned that AI is not there to replace the therapist, due to the perception that this new technology can start replacing the role of the therapist. In a recent study, researchers found that in 20 scenarios, AI had increased emotional awareness compared to the general population—leading to the fear of replaced jobs [24]. For instance, in comparison to traditional mental health sessions, conversational agents can lead to improved control, choice, and interactivity over session content. While AI can be used for supplementally guide therapy sessions, practitioners cannot be solely relied for delivering therapy [33]. Current use of generative AI for conversational agents in mental health, as used in therapy sessions, has contradictory outcomes. Heston (2023) found that when used on simulated patients, generative AI-based conversational agents are slow to escalate mental health risk scenarios, postponing referral to a human to potentially dangerous levels [40].

There are many applications of AI in mental health that are very positive as well. However, other researchers have found a potential improvement by applying conversational agent AI, i.e., chatbots, to capture dynamic human behavior adequately to provide responses tailored to users’ personalities [41]. Researchers in the field of human–machine interaction stressed the importance of avoiding “one-size-fits-all” interactions. Völkel et al. found approaches to adapt a voice assistant’s personality to the user, improving the interactive experience [42]. This adaptive automated user experience can prove to be a useful application of AI.

### 4.1. Limitations

There are several limitations of this study. First of all our study is based on Google Trends data and there is no way to assess how accurately these data represent the general population, potentially affecting the reliability and generalizability of the results. However, predictive models using this method have been applied by other researchers, reflecting the general population. We also did not assess the fears and concerns people have about AI. More specifically, as AI applications in mental health increase, so too does the likelihood of cybersecurity attacks of confidential mental health information. We did not research how people’s fear may be driving some of the research. However, even negative perceptions can equate to some of the trepidation that people feel about the novel technology. Currently, Amazon and Microsoft are in an AI war to create the most optimal platform. However, news and social media can erroneously deflect focusing on mental health and not just news. Patient information must remain ethically confidential, leading to further concerns within the mental health industry about the unbridled growth of generative AI. These areas require further research. To mitigate these risks, Habbal et al. demonstrated the effectiveness of an important framework.

We did not study the impact of certain systematic approaches to understanding AI. Further innovative approaches should be explored in future studies. One such approach is to conduct AI risk management. Artificial Intelligence Trust [43], Risk and Security Management (AI TRiSM) is a systematic approach to manage risks of AI. Applying the discipline of mental health, future studies should explore the effectiveness of frameworks to mitigate such risks of releasing sensitive information. Furthermore, bias is another serious threat against creating optimal AI models. AI applications will not mitigate mental health disparities if they are built from historical data that reflect underlying social biases and inequities [44]. In mental health applications, AI-related hallucinations, due to biases in the data, inadequate training, or flawed algorithms, can also be a potential negative byproduct of overreliance on this technology.

Another limitation is that we did not study the context of training data and how this raises major concerns in the spread of AI. Biases can be mitigated through increased use of LLM training data that are more diverse in mental health. According to Kuzlu et al., with the proliferation of Internet of Things (IoT) through wearable devices, AI is becoming more popular [45]. However, cyberattackers are beginning to exploit the weaknesses of AI, known as generative adversarial AI, to carry out cybersecurity attacks [46,47,48,49]. The types of attacks can be categorized as poisoning (AI training data being intentionally tampered), evasion, extraction, and inference—slowing down AI adoption in the area of mental health [50,51]. However, not all adversarial AI is harmful, as this can also be used to train and leverage neural networks in guiding mental health treatment and prevention. Finally, another limitation is that there was no indication whether some of the popularity was due to mistrust or interest, as the two are conflated in using Google Trends. The rapid growth in AI necessitates an improved understanding of how cyberattacks can influence the popular opinion and trepidation of the public about AI.

### 4.2. Recommendations

There are multiple recommendations that can aid in integrating AI with mental health services. With initial integration of AI in the mental health field, there can be further enrichment of training data, making the application more accurate and less biased. This can help alleviate some of the burden that is experienced by general health practitioners. One application that researchers have used is synthesizing attack trees using LLMs to predict cyberattack scenarios, potentially jeopardizing medical privacy in mental health, thereby fostering mistrust. Most recently, the AI Digital Bill of Rights has been established to ensure safe and effective systems, data privacy, and algorithmic discrimination protections [52,53,54,55]. In the following section, we outline some of the important recommendations in applying AI in mental health.

- Increasing AI awareness among the general public will fuel the transition from traditional therapeutics to AI-assisted therapeutics in the area of mental health that practitioners can act on.
- Improve health literacy about the understanding of the mental health condition to know more about what they are experiencing in LLM, and elevating this.
- Increase dynamic interplay between humans and AI rather than replacing healthcare practitioners, leveraging the strengths of each.
- Differentiate between AI detection of physical and mental health problems that are similar, such as atrial fibrillation versus anxiety.
- Increase the use of AI gradually to address gaps created from the mental health profession shortage.
- Apply potential advancement and application of AI in mental health sectors, by using AI-based tools to empower patients.

## 5. Conclusions

With the growth of large language modeling, mental health practitioners and patients alike must be well informed about this powerful tool’s vast applications. Our study demonstrates the increasing awareness of mental health and AI among the general public, making advocacy and education about AI technology of paramount importance. Not only is this modality effective for diagnostic purposes, but there are also treatment applications that have mostly been untapped. Some treatment modalities include automated cognitive behavioral therapy and finding medication regimens that are most effective for the mental health condition within the individual. Future popularity trends in the discipline of mental health and topics like depression and anxiety are predicted to increase in popularity. As mental health diagnostic, treatment, and prevention approaches become more accurate, there is a need to apply novel technologies such as AI to increase the diagnostic precision and accuracy. With the rapid growth of AI in mental health, care must be taken to protect confidentiality from cyberattacks and potential bias that may arise from the application of AI. Most importantly, our study shows how AI is perceived by the general public, driving attitudes and uptake of this novel technology, by both mental health providers and patients.

## Figures and Tables

**Table 1:** The minimum and maximum relative search volume (RSV) for each search term and the corresponding dates.

Google Trends Search Term	Date	RSV Min/Max
AI	1 January 23	28 (min)
	16 April 23	100 (max)
AI and Mental Health	22 January 23	19 (min)
	15 October 23	100 (max)
AI and Depression	17 December 23	23 (max)
	12 November23	100 (max)
AI and Anxiety	8 January 23	17 (min)
	26 November23	100 (max)

**Table 2:** Predicted relative search volume in “AI and Mental Health” search terms from January 2024 to December 2024.

Month	Relative Search Volume
January 2024	25.5
February 2024	27.7
March 2024	19.5
April 2024	19.3
May 2024	26.7
June 2024	31.8
July 2024	29.7
August 2024	35.5
September 2024	39.7
October 2024	47.7
November 2024	49.2
December 2024	54.6

## Data Availability

The data are stored in the following repository https://github.com/skbanergt/AI (accessed on 2 July 2024).
